# Everybody's Page

**Published:** 1892-01-23

**Authors:** 


### Everybody's Page.

Salol, which is freely soluble in ether, has recently been suggested as a good coating material for pills.

So many discoveries have been made during the past fifty years that people are beginning to cease being surprised at man claiming any fresh power over nature. According to a German journal, a Moscow dentist can grow teeth for us. If this enterprising gentleman would only grow painless teeth for us at the outset, and save us constant agonies from birth to death, he would not only prove a benefactor to the human race, but to his own pecuniary welfare. At present, however, he confines his attention to growing new teeth on the ruins of old ones, which are said to grow as firmly into the gums as natural ones Even this advance in dentistry will be good news to those who have to wear false teeth, which insist upon falling into the lap of the proud owner just as she wishes to impress upon a rival how captivating her row of white pearls makes her.

It is a vulgar belief in Mexico that croup was unknown in that country before the intervention of Napoleon III. in 1862-1867. This belief has probably arisen from the fact that during the past twenty years there has been a considerable increase in this disease. It is also generally supposed that the inhabitants of the table-land are quite free from consumption; whether this is so or not, that disease is the cause of a large percentage of mortality in the City of Mexico.

Preserved fruits are now eaten on so large a scale, that caution should be used in their consumption. Though glass and porcelain vessels are used much more frequently now than was the case a few years ago, tins are still used to a very considerable extent. In most cases tinned fruits should be avoided, for there will often be found to exist in the fruits so preserved metallic impurities which are likely to endanger the health. No fruits containing acid juices should be preserved in anything but glass or earthenware, as has recently been pointed out by the Medical Officers of Health for Marylebone, and if people are offered such fruit in tins they will be wise to decline them.

